# A Statement of the Causes Which Led to the Dismissal of Surgeon General William A. Hammond from the Army; with a Review of the Evidence Adduced before the Court

**Published:** 1865-03

**Authors:** 


### BOOKS REVIEWED.

*A Statement of the Causes which led to the Dismissal of Surgeon General William A. Hammond from the Army; with a Review of the Evidence adduced before the Court.*

The above is the title of a pamphlet issued by Dr. Hammond in his own vindication, and which has been awaiting notice at our hands for some time. Of course all our readers are familiar, from the newspapers, with the general facts of Dr. Hammond's arrest, trial, and condemnation, but probably comparatively few of them will see, or have seen, his pamphlet.

We have perused this production carefully, and if we are not fully prepared to pronounce Dr. Hammond guiltless of the charges preferred against him, we are fully prepared to pronounce the moral atmosphere of Washington very bad. If the Doctor has not succeeded in convincing his readers that his honor and integrity are unimpeachable, in face of the findings and sentence of the Court which tried him, he has at least fully established the existence of an organized system of fraud, corruption, and dishonesty, perfectly appalling to the mind of a quiet country doctor, not to mention the minor sins of lying, brow-beating, and the like.

Dr. Hammond argues his case with great shrewdness and ability before the bar of public opinion. He seeks to convince his readers that the majority of the Court Martial which tried him were corruptly influenced against him, that especially the Judge Advocate, (the most powerful member of a Court Martial) was detailed expressly to convict him, and that all these corruptions, and the still more corrupt Commission which made the preliminary investigations in his department, were set in motion by the hatred of Mr. Secretary Stanton, who did not like the doctor. "The cause" for this hatred, he tells us on page 16, "existed in the fact that I gave him to understand from a very early period of my official career, that I, for one, would not quietly submit to the insolence which he constantly exhibited toward his subordinates."

Now we know something of the fallibility of the judgments of Courts Martial, as well as of the immense power wielded by a prejudiced Judge Advocate, but the Doctor must excuse us if we are not inclined in the face of the *status quo*, to accept his *ex parte* statement or argument, in opposition to the decree of a court which may have been corrupt, but which was presumably fair. And we must doubt, although the Doctor was undoubtedly

"Multum et terris jactatus et alto,"

whether it was due solely to the "memorable wrath" of the cruel Stanton.

But whatever be the Doctor's condition, whether of guilt or innocence, (we sincerely hope the latter,) time will sooner or later reveal it. If what he says be entirely true, we have a picture of corruption from which our suffering people may recoil in utter amazement; if it be only partially true, we still have a shameful exhibition.

We are very much afraid for the credit of our people, that his pictures of Mr. Stanton's overbearing petulance (to use no harsher word) are not overdrawn, and we must condemn as gentlemen and professional men such treatment as is there depicted of the highest representative of our calling in the army, whatever be that representative's character. But space forbids our doing this branch of the subject justice, and we must postpone it.

Let us be understood as saying, then, with the kindest disposition towards Dr. Hammond, that, if we do not at once pronounce him innocent, we are certainly not fully convinced of his guilt; and if ever so guilty, he has shown himself "no worse than his neighbors." For our profession's sake, we hope his entire innocence may yet appear. P.

